# Changes in the Sensitization Pattern to *Alternaria alternata* Allergens in Patients Treated with Alt a 1 Immunotherapy

**DOI:** 10.3390/jof7110974

**Published:** 2021-11-16

**Authors:** David Rodríguez, Ana I. Tabar, Miriam Castillo, Montserrat Martínez-Gomariz, Isabel C. Dobski, Ricardo Palacios

**Affiliations:** 1 Research and Development Department at Diater Laboratorios, 28919 Madrid, Spain; m.castillo@diater.com (M.C.); m.martinez@diater.com (M.M.-G.); i.dobski@diater.com (I.C.D.); r.palacios@diater.com (R.P.); 2 Spanish Research Network on Asthma, Adverse Drug Reactions, and Allergy (ARADyAL) of the Carlos III Health Institute of Madrid, 28029 Madrid, Spain; ana.tabar.purroy@navarra.es; 3 Allergology Service, Hospital Complex of Navarra, 31008 Pamplona, Spain

**Keywords:** Alt a 1, immunotherapy, *Alternaria alternata*, sensitization pattern, immunoblotting, allergen, fungal allergy

## Abstract

*Alternaria alternata* is the most important allergenic fungus, with up to 20% of allergic patients affected. The sensitization profile of patients sensitized to *A. alternata* and how it changes when treated with immunotherapy is not known. Our objective is to determine the allergen recognition pattern of allergic patients to *A. alternata* and to study its association to the parameters studied in a clinical trial recently published. Sera of 64 patients from the clinical trial of immunotherapy with native major allergen Alt a 1 were analyzed by immunoblotting; 98. 4% of the patients recognized Alt a 1. The percentage of recognition for Alt a 3, Alt a 4, and/or Alt a 6, Alt a 7, Alt a 8, Alt a 10 and/or Alt a 15 was 1.6%, 21.9%, 12.5%, 12.5%, and 12.5% respectively. Of the 64 patients, 45 (70.3%) only recognized Alt a 1 among the allergens present in the *A. alternata* extract. Immunotherapy with Alt a 1 desensitizes treated patients, reducing their symptoms and medication consumption through the elimination of Alt a 1 sensitization, which is no longer present in the immunoblotting of some patients. There may be gender differences in the pattern of sensitization to *A. alternata* allergens, among others.

## 1. Introduction

The genus *Alternaria* belongs to the subclass of *Hyphomycetidae*, Fungi imperfecti, and is characterized by septate hyphae and reproduction by multicellular conidia, called pheodictic dictyospores. The conidia are transversely septate and acquire the appearance of mace, forming long chains. The species of the genus *Alternaria* present colonies of filamentous appearance and gray, brown, or black colors [1]. The saprophytic character and the ability to adapt to a wide range of climatic conditions allow a universal distribution and development, both outdoors and indoors, especially in hot and dry climates, where, at the end of summer, atmospheric counts can exceed 7000 conidia/m^3^, with 300/m^3^ indoors [2]. A range between 80 and 300 conidia/m^3^ has been established as the threshold concentrations of *Alternaria* capable of eliciting allergic symptoms [3]. Currently, more than 50 different species belonging to the genus *Alternaria* have been isolated [4] 

*Alternaria alternata*, initially named *Torula alternata*, Fries 1832, also known as *Alternaria tenuis,* Simmons 2007, is the most cosmopolitan representative of the genus as well as one of the main atmospheric pollutants. It is a source of IgE-mediated allergen sensitization and is related to the development and exacerbation of respiratory disease conditions such as asthma [2], rhinosinusitis [5], and allergic bronchopulmonary mycosis (ABPM) [6], as well as the so-called hypersensitivity pneumonitis, also known as extrinsic allergic alveolitis [7].

The prevalence rates of *A. alternata* sensitization are highly variable depending on the geographical area, the population included, and the diagnostic method used. In Europe, it is estimated to be between 4.4% [8] and 9% [9] of the general adult population, and, in the US, the prevalence rate is estimated to be as high as 12.9% [10]. Recently, our working group published that the prevalence of sensitization to *A. alternata* in Spanish allergic patients has reached 18.4% [11]; this is close to the 20% described previously [12].

To date, up to 17 allergens [13] have been described for *A. alternata*, of which 12 are listed by the IUIS [14]. Alt a 1 is a 29 kDa dimer of unknown biological function and high structural stability that localizes to spore walls [15]. It is considered to be the major allergen, with a prevalence of sensitization between 80% and 98% of the population allergic to *A. alternata* [12]. It is the primary marker of sensitization to the fungus and is mainly responsible for the symptoms shown by patients [16]. This allergen is not exclusive to the genus *Alternaria* but has also been described in other genera of the *Pleosporaceae* family, such as the genera *Ulocladium* and *Stemphylium,* which show a high degree of cross-reactivity [17,18]. The other allergens described are considered minor in terms of the percentage of patients with specific IgE against them and with less relevance in allergic disease than Alt a 1. Among these are Alt a 3 (heat shock protein) with 16 kDa [19,20], Alt a 4 (disulfide isomerase) with 57 kDa [21,22], Alt a 5 (P2 protein) with 11 kDa [20,23], Alt a 6 (enolase) with 47 kDa and high cross-reactivity with the other fungal enolases [24,25,26], Alt a 7 (YCP4 protein family) with 22 kDa [21,22], Alt a 8 (NADP-dependent mannitol dehydrogenase) with 28.6 kDa [27], Alt a 10 (aldehyde dehydrogenase) with 11 kDa [21,22], Alt a 13 (glutathione-S-transferase) with 26 kDa [28,29], Alt a 14 (manganese-dependent superoxide dismutase) with 24 kDa [26,30,31], and Alt a 15 (serine protease) with 58 kDa [32,33]. These minor allergens have a recognition rate by patients sensitized to *A. alternata* of between 2% and 42%.

Several studies describing the relevance of the different allergens from the main sources of sensitization, such as grass pollen [34], tree pollen [35], mites [36], epithelia [37], and *Hymenoptera* venom [38], from the point of view of sensitization and IgE recognition by patients have recently been published. However, in the case of fungi, specifically for *A. alternata*, there are no such studies with an adequate number of patients.

We analyze the sera of 64 patients sensitized to *A. alternata* and Alt a 1 to study the pattern of allergen recognition and its relationship with the demographic variables of the population, their baseline pathology related to rhinitis and asthma, and the efficacy and safety variables obtained in the first clinical trial conducted with molecular immunotherapy with the major allergen Alt a 1 in its native form [39]. Being able to use the same sera from patients enrolled in the clinical trial allows us to obtain more in-depth and robust information on how the pattern of allergen sensitization affects the efficacy and safety of allergen immunotherapy.

## 2. Materials and Methods

### 2.1. Patients and Serum Analysis

The patient data shown as well as the serum samples analyzed in this study belong to an ad-hoc analysis from data and sera obtained in a multicenter, randomized, double-blind, parallel-group placebo-controlled clinical trial of distinct concentrations of Alt a 1. The trial was authorized by the Clinical Research Ethics Committee, Hospital Complex of Navarra, Spain, and the Spanish Agency for Medicines and Health Products under the heading Eudra CT 2010-0244015. The trial was initiated in 2012 and concluded in 2016. These data were published in J Allergy Clin Immunol. 2019. The serum of 64 patients belonging to the population Per-Protocol (PP) was analyzed for this study. All patients signed the informed consent form, which included the possibility of carrying out analyses and obtaining additional data after the conclusion of the clinical trial for which they had been selected.

### 2.2. SDS-PAGE and Immunoblotting

An extract of *Alternaria alternata* from strain 103.33 was obtained by double water-soluble extraction and subsequent freeze-drying.

Proteins from the *A. alternata* extract were analyzed by SDS-PAGE, according to the Laemmli method, in 15% polyacrylamide gels under reducing conditions. Proteins were visualized by silver staining (Thermo Scientific, Waltham, MA, USA) and transferred to polyvinylidene difluoride membranes (PVDF, Trans-blot turbo TM. Bio-Rad, Hercules, CA, USA).

The binding of IgE antibodies to allergens was analyzed by immunoblotting using individual patients’ sera and anti-human IgE peroxidase conjugate (Southern Biotech, Birmingham, AL, USA). Chemiluminescence detection reagents (Western lightning^®^ Plus-ECL, Perkin Elmer, Waltham, MA, USA) were added, following the manufacturer’s instructions.

The strength of IgE binding to the Alt a 1 protein was measured using Image Lab software version 5.2.1 build 11 (Bio-Rad, Hercules, CA, USA). In all cases, the relative intensity of the Alt a 1 band at baseline time in each patient was considered as a reference band and, therefore, as 100% IgE binding (relative quantification = 1). Finally, at successive times (Year 1 and Year 2), the intensity of the Alt a 1 band was compared with the intensity at baseline.

### 2.3. Digestion and Shotgun LC-MS Analysis

Proteomic analysis was performed in the Proteomics Unit of the Complutense University of Madrid, a member of ProteoRed, and supported by grant PT17/0019 of PE I+D+i 2013–2016, funded by ISCIII and ERDF.

Peptides digest from the *A. alternata* extract were analyzed by RP-LC-ESI-MS/MS in an EASY-nLC 1000 System coupled to the Q-Exactive HF mass spectrometer through a Nano-Easy spray source (all from Thermo Scientific).

Then, 1 µg of peptides was loaded first onto a pre-column Acclaim PepMap 100 Trapping column (Thermo Scientific, 20 mm × 75 μm ID, 3 μm C18 resin with 100 Å pore size) and then separated and eluted on an analytical reverse-phase Easy Spray column (Pepmap RSLC C18n 500 mm × 75 μm ID, 2 μm C18 resin with 100 Å pore size) with an integrated spray tip. A 150 min gradient of 2% to 35% Buffer B (100% acetonitrile, 0.1% formic acid) in Buffer A (0.1% formic acid) at a constant flow rate of 250 nl/min was used for the elution of peptides.

Data acquisition was performed with a Q-Exactive HF using data-dependent acquisition (DDA) and in positive mode with Xcalibur 4.0 software. From each Full MS (350–1800 Da) scan, the top 15 most abundant precursors, with charges of 2–6 in MS 1 scans, were selected for higher-energy collisional dissociation (HCD) fragmentation with a dynamic exclusion of 20 s.

### 2.4. Protein Identification

Peptide identification from raw data was carried out using the Mascot v. 2.6 search engine through Protein Discoverer 2.4 software (Thermo Scientific). A database search was performed against UP-FUNGI (11599317 sequences, 23 February 2020) from the UNIPROT (www.uniprot.org (accessed on 6 April 2021)) database. Using the Sequest HT search engine, the database used for searching, all *A. alternata* allergens were annotated in Allergome (www.allergome.org (accessed on 6 April 2021)). A contaminant database (247 sequences) was included in both databases.

The following parameters were used for the searches: tryptic cleavage after Arg and Lys, up to two missed cleavage sites allowed, and tolerances of 10 ppm for precursor ions and 0.02 Da for MS/MS fragment ions; the searches were performed allowing optional methionine oxidation and fixed carbamidomethylation of cysteine. A search against a decoy database (integrated decoy approach) was used to calculate the FDR. The acceptance criteria for protein identification are an FDR < 1% and at least one peptide identified with high confidence (CI > 95%).

### 2.5. Statistical Analysis

Contingency tables have been used for the analysis of the data for the comparison of two or more proportions using the Z test or the chi-square test, as appropriate. The interval of confidence was 95% for all tests. *p*-values less than 0.05 were considered statistically significant. Data management and graphical representation were made using the GraphPad Prism statistical package version 9.1.2.226 for Windows (GraphPad Software, La Jolla, CA, USA).

## 3. Results

**Figure 1:**
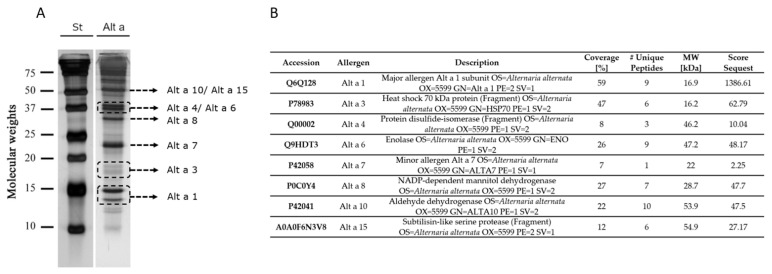
Silver SDS-PAGE under reducing conditions of the allergenic extract of *A. alternata*. St: molecular weight marker. Alt a: allergenic extract of *A. alternata*. (**A**). Allergen Identification of SDS-PAGE bands by liquid–mass spectrometry (LC-MS). Accession number, description, and molecular weight are reported by the NCBI database. Score and % of sequence covered by identified peptides are reported by mascot result (**B**).

The allergenic extract of *A. alternata* was obtained using collection strain 103.33., being the same strain used for the purification and isolation of Alt a 1 for its use in molecular immunotherapy of patients sensitized to *A. alternata*. The presence of the main allergens described for *A. alternata* in the allergenic extract was determined by LC-MS. Once each of the proteins had been identified, they were assigned their identity in the SDS-PAGE performed with the allergenic extract of *A. alternata* (Figure 1A,B) by the similarity of molecular weights between those obtained in LC-MS identification and electrophoresis under reducing conditions.

Sera from patients in the PP population participating in the clinical trial of immunotherapy with native Alt a 1 [39] were used for immunoblotting with the allergenic extract of *A. alternata*. The demographic data of the study population can be seen in Table S1.

**Figure 2:**
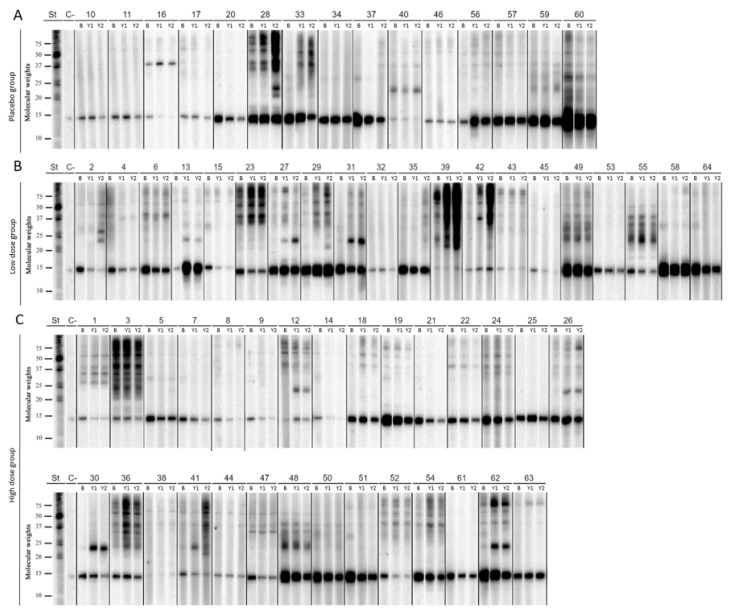
Immunoblotting performed with the allergen extract of *A. alternata* using patients’ sera from placebo (**A**), low dose (**B**), and high dose group (**C**). St: molecular weight marker; C−: negative control; B: baseline; Y1: Year 1; Y2: Year 2.

Of the 64 sera analyzed by immunoblotting (Figure 2 and Table S2), 70.3% (45) recognized only Alt a 1 before starting immunotherapy treatment with Alt a 1 (baseline). All patients, except for one, had an Alt a 1 sensitization band, which represents 98.4% of the patients; 1.6% had specific IgE against Alt a 3, 21.9% recognized Alt a 4 and/or Alt a 6, 12.5% showed sensitization to the Alt a 7 protein, and the same percentage of patients showed sensitization to Alt a 8 and Alt a 10 and/or Alt a 15 (Table 1).

After one year of immunotherapy with Alt a 1, there were patients whose recognition of the Alt a 1 band was below the intensity of the negative control, for example, or alternatively, with a decrease in their sensitization to Alt a 1 below the detection limit of the technique. Specifically, 6.7% of patients in the placebo group (these patients did not receive immunotherapy during the first year), 10% of patients in the low-dose group (0.2 µg Alt a 1/dose), and 6.9% of patients in the high-dose group (0.37 µg Alt a 1/dose) had no Alt a 1 recognition. After the second year of immunotherapy with Alt a 1, the percentage of patients who do not recognize Alt a 1 was 13.3% in the placebo group (this group receives the 0.37 µg Alt a 1 dose during the second year of the clinical trial), 20% in the low dose group, and 17.3% in patients in the high dose group (Figure 2 and Table 1).

The influence of the demographic and clinical baseline characteristics of the study population on their sensitization to *A. alternata* allergens by immunoblotting was studied. As regards the age group of the patients, it was observed that adolescents (12 to 18 years old) in the placebo group showed a lower sensitization to Alt a 7 during the first year of the trial; 0% compared to 22.2% of adults. Adolescents in the placebo group also showed a higher rate of sensitization to Alt a 4 and/or Alt a 6 during the first year and to Alt a 10 and/or Alt a 15 during the first and second year of the trial compared to the placebo group in the total and adult populations. Adolescents in the low-dose group had a higher % sensitization to Alt a 10 and/or Alt a 15; 37.5% compared to 8.3% in the adult population. Finally, adolescents in the high-dose group presented a higher sensitization rate to Alt a 7, both in the first and second year of the trial, being, in both cases, 41.7% versus 17.6% in the adult population (Figure 2 and Table 1).

Regarding the gender of the population, it was observed that males had a higher percentage of polysensitized patients compared to the total population and females; 37.8% compared to 18.5% of females. This difference explains why females, with 81.5% of patients monosensitized to Alt a 1, have a lower rate of recognition of Alt a 4 and/or Alt a 6, Alt a 7, Alt a 8 and Alt a 10, and/or Alt a 15 than males and the total population, both at baseline and during the two years of the trial. On the other hand, males in the low-dose group have higher % sensitization to Alt a 4 and/or Alt a 6 and Alt a 8 during both years of the trial compared to both the total population and females. Finally, both females in the low and high dose groups had a much higher % of patients who did not recognize Alt a 1 at the end of the trial than males and the total population, being 27.3% in the case of females in the low dose group and reaching 36.4% in the high dose group.

Regarding the severity of rhinitis at baseline, graduated according to the ARIA criteria, patients with mild persistent rhinitis have a higher percentage of polysensitization than the total population, reaching 38.5%. The mild persistent rhinitis group had a higher rate of recognition of Alt a 8 and Alt a 10 and/or Alt a 15 in both the first and second year of the trial compared to the other rhinitis groups and to the total population, with 50% in both cases for the two years of the clinical trial. This same population, after the end of the second year of the clinical trial, also showed a higher % of recognition of Alt a 3, Alt a 4 and/or Alt a 6, and Alt a 7.

Regarding asthma, graduated according to the GINA criteria, patients with intermittent asthma presented a rate of 50% of polysensitization, which is higher than in the total population and the rest of the asthma classification groups. This means that within the placebo group of this population, all patients recognized Alt a 4 and/or Alt a 6, Alt a 8 and Alt a 10, and/or Alt a 15 during the two years of the trial, this rate being higher than in the total population and the rest of the asthma classification groups. In addition, within this group, all patients recognize Alt a 3 and Alt a 7 after the second year of the clinical trial, being higher than the percentage shown for these allergens by the total population and the rest of the asthma classification groups. Patients with intermittent asthma belonging to the low dose group also recognized Alt a 7 after the second year of the clinical trial, with a higher percentage (71.4%) than the rest of the asthma classification groups. In the population with moderate/severe persistent asthma, both in the low-dose group and in the high-dose group, the percentage of patients who did not recognize the Alt a 1 allergen after the second year of immunotherapy was 42.9% and 41.7%, respectively, being a higher % than those shown by the total population and by the rest of patients with other asthma classifications.

**Figure 3:**
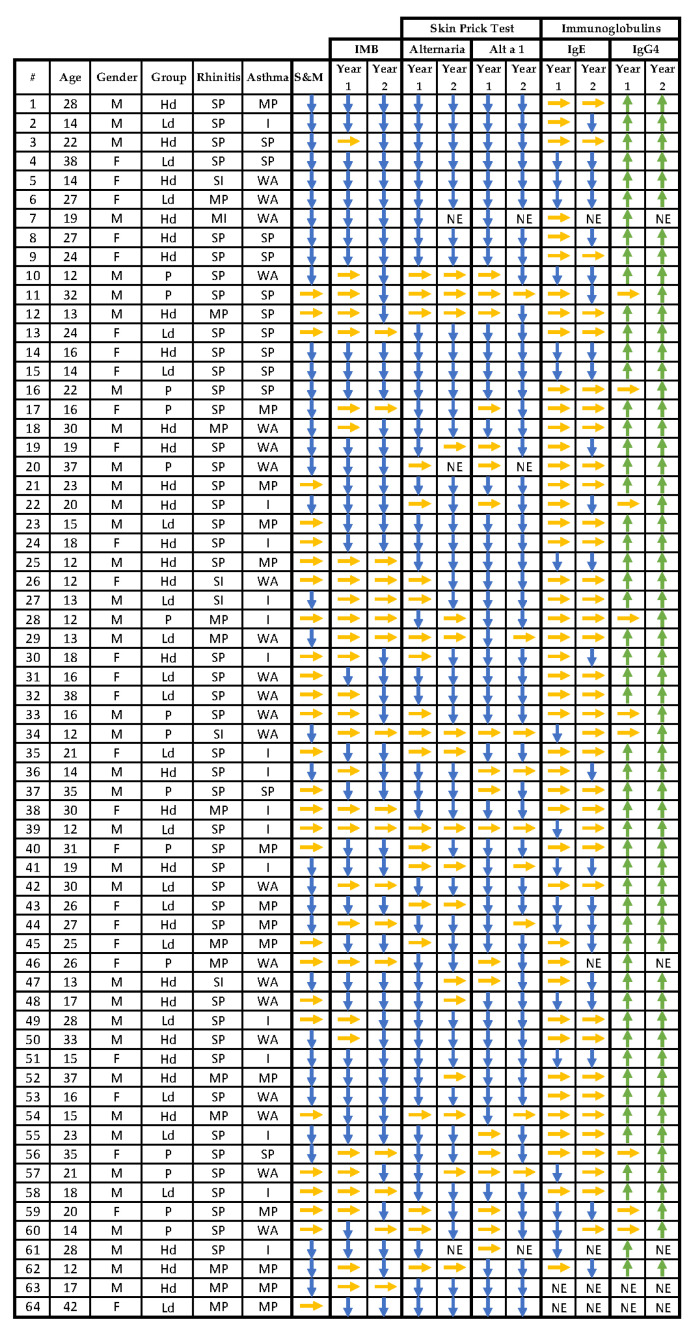
Demographic and clinical characteristics and behavior in the efficacy variables of the clinical trial compared to baseline. A 20% reduction (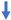) compared to baseline in the symptoms score and medication consumption, the intensity of the Alt a 1 band on immunoblotting, the area of wheal produced by the complete extract of *A. alternata*, and Alt a 1 and IgE concentrations is considered an improvement compared to baseline. A 20% increase in IgG4 antibody concentration is considered an improvement (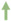). If there is no reduction or increase of 20%, respectively, no improvement is considered (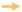). M: male; F: female; P: placebo; Ld: low dose; Hd: high dose; MI: mild Intermittent; SI: severe Intermittent; MP: mild persistent; SP: moderate/severe persistent; WA: without asthma; I: intermittent; NE: no sample available; IMB: immunoblotting.

In a post-hoc analysis of the results of the clinical trial performed [39], the correlation between the data obtained from the clinical trial and the presence of the different *A. alternata* allergens in the immunoblotting was analyzed. (Table 2 and Figure 3). Data shown in the clinical trial [39] was confirmed as regards the high-dose group after the first year of immunotherapy with Alt a 1, having higher percentages of improvement than those shown by the placebo group for the variables of symptom score and medication consumption, wheal area with *A. alternata,* and Alt a 1 SPTs and IgG4 against Alt a 1. This trend is also maintained in the % of patients who decrease the intensity of the Alt a 1 band on immunoblotting. The low dose group also had a higher % of patients than the placebo group in which the intensity of the Alt a 1 band decreases after the first year of the clinical trial. In the second year, the placebo group was no longer available (which was then treated with the high-dose product), and it was observed that in all three groups, the percentage of patients who improved in their variables was higher than that shown in the first year of the clinical trial, analogous to what was observed in the original analysis of the trial.

In the analysis by age group, adolescents in the placebo group had a higher percentage of patients who decreased the intensity of the Alt a 1 band in the immunoblotting with respect to adults. Adolescents in the high-dose group had a higher percentage of decrease in IgE than adults; 41.7 vs. 17.6%.

Females in the placebo group who received the high dose in the second year of the trial reduced the area of wheals produced by *A. alternata* and Alt a 1 SPT, being higher than that shown by males; 40% and 60%, respectively. Females in the low dose group reduced the intensity of the Alt a 1 band on immunoblotting over the two years of the trial by a higher percentage than males. Females in the high-dose group showed a higher percentage reduction in *A. alternata* wheal area and IgE reduction than males.

All patients with mild persistent rhinitis in the placebo group had a higher reduction in *A. alternata* wheal area than that shown by the two rhinitis groups in the study. Patients in the high-dose group of this same population showed a lower percentage of reduction in IgE than that shown by the total population and the other two rhinitis categories in both years of the clinical trial. Patients in the placebo group of the moderate/severe rhinitis population reduced the intensity of the Alt a 1 band in the immunoblotting by 41.7% in contrast to 0% in the two other rhinitis groups.

A higher percentage of patients without asthma in the placebo group in the first year decreased their symptoms and medication consumption score (S&M) with respect to the rest of the asthma groups. The same trend was seen in the decrease of IgE, which reached 57.1% in this group. Within the low-dose group of patients without asthma, a greater number of patients decreased their S&M score with respect to the other two asthma groups. Finally, patients with moderate/severe persistent asthma in the low-dose group had decreased IgE levels compared to the other two asthma groups.

The sensitization pattern to *A. alternata* allergens of the patients who developed systemic adverse drug reactions (ADRs) during the clinical trial was analyzed (Table 3). No factors were found to be associated with the occurrence of systemic ADRs, either related to the pattern of allergen recognition or to the severity of rhinitis and asthma.

## 4. Discussion

The post-hoc analysis performed in this study on the sera of patients from the PP population of the clinical trial of immunotherapy with Alt a 1 [39] allows us to complement the information obtained on the response of patients to molecular immunotherapy with a single allergen, Alt a 1. The data obtained in this study also complete the information available on the pattern of recognition of *A. alternata* allergens in patients sensitized to the fungus. The prevalence values for *A. alternata* allergens obtained in this study are similar to those previously described in the literature and show that the allergen recognized by the vast majority of patients (98.4%) is Alt a 1 [12], the main cause of the symptoms of allergy mediated by sensitization to *A. alternata* [16]. In addition, 70.3% of patients recognized only one allergen within the complete extract of *A. alternata*, which was Alt a 1. This data confirms the role of Alt a 1 in patients sensitized to the fungus and makes it a unique model to compare with the rest of the allergenic sources; patients usually recognize multiple allergens even if the major allergens are the relevant ones [40].

In general, during treatment with Alt a 1 immunotherapy, patients tend to become moderately sensitized to the other allergens of *A. alternata* because exposure to the fungus continues and sensitization to Alt a 1 decreases or even disappears. After receiving immunotherapy with Alt a 1 for two consecutive years, up to 20% of the patients had no recognition of Alt a 1 on immunoblotting, confirming the desensitizing effect of Alt a 1 immunotherapy. This effect is directly related to the negativization of skin tests observed in the clinical trial with *A. alternata* and Alt a 1 pricks in some patients [39]. These data may explain one possible mechanism by which patients reduce their symptoms and symptomatic medication consumption when treated with Alt a 1 immunotherapy—it is due to their desensitization to the Alt a 1 allergen, which, in some patients, is no longer present in their immunoblotting at the end of immunotherapy treatment. This behavior was especially accentuated in females, with up to 36.4% of females not recognizing the Alt a 1 band after the end of the trial, and in patients with severe moderate persistent asthma, with up to 42.9% of patients not recognizing the Alt a 1 band after the end of the trial.

Regarding the efficacy variables analyzed post-hoc, the parameter of reduction of Alt a 1 band intensity on immunoblotting correlates with the improvement of S&M and the reduction of wheals with *A. alternata* and Alt a 1 SPT. Females responded better to Alt a 1 immunotherapy, considering that they were mostly monosensitized to Alt a1, as they reduced the area of wheals with *A. alternata* and Alt a 1 SPT, IgE, and the Alt a 1 band on immunoblotting to a greater extent than males.

In our population, we found prevalence rates for minor allergens similar to those reported in the bibliography, with the only exception of Alt a 8, which, in our study, was recognized by 12.5% of patients compared to the 41% reported in previous studies [27]. Adolescents showed a higher percentage of sensitization to Alt a 4/6, Alt a 7, and Alt a 10/15 during immunotherapy treatment. However, females showed lower percentage recognition of Alt a 4/6, Alt a 7, Alt a 8, and Alt a 10/15. Patients with mild persistent rhinitis showed higher prevalence rates for Alt a 3, Alt a 4 and/or Alt a 6, Alt a 7, Alt a 8, and Alt a 10/15. All patients with intermittent asthma in the placebo group recognized all *A. alternata* allergens at the end of the clinical trial. Males and patients with mild persistent rhinitis and intermittent asthma had a higher percentage of patients recognizing more than one allergen within the study.

It should be noted that the prevalence data of the different *A. alternata* allergens obtained in this study are conditioned by the population from which they come, as all prevalence studies are. However, in this case, the population comes from a clinical trial [39] where one of the inclusion criteria was that patients had a positive SPT for both *A. alternata* and native Alt a 1. Only 5% of patients had to be rejected (screening failure) because they were negative for Alt a 1 SPT, so we consider that this situation does not affect the results obtained.

On the other hand, in our study, the allergens are native, forming part of the complete allergenic extract of *A. alternata*, being fully representative of the process when the patient is exposed to the allergenic source, either outdoors or indoors. This is in contrast with the prevalence data obtained from other studies, which are obtained by cloning the allergen and obtaining the recombinant form. Immunoblotting allows us to know the complete sensitization profile of a patient and to analyze the influence of each individual allergen on the severity of the pathology, the efficacy of immunotherapy treatments, and the likelihood of ADR occurrence [36,38]. In the case of *A. alternata*, it is the only technique available that allows us to study the patient’s complete allergen profile since commercial platforms for specific IgE analysis are only available in their recombinant forms, rAlt a 1 (ImmunoCAP and ALEX) and rAlt a 6 (ALEX).

In the characterization of our allergenic extract of *A. alternata*, 8 of the 12 allergens described for *A. alternata* in IUIS were identified; missing were Alt a 5, Alt a 12, Alt a 13, and Alt a 14. However, in the extract used, we observed bands between 10 and 15 kDa (Figure 1) that could be compatible with Alt a 5 and Alt a 12 and could not be identified due to their low quantitative presence in the fungus. In no case were bands found in the immunoblotting above 15 kDa, so their non-identification does not affect the results obtained. As for Alt a 13 and Alt a 14, no bands were found in the allergenic extract in the range of 24–26 kDa, and they could be masked by Alt a 7 (22 kDa), which was much more present in quantity in the fungus [13]

In patients who developed Grade 1 and/or Grade 2 systemic ADRs, no parameter associated with the occurrence of these was found. It has been reported in the literature that in patients sensitized to minor allergens of *A. alternata,* there could be a higher risk of developing severe systemic ADRs [41]; however, in our case, we did not find this result, which was influenced by the fact that the immunotherapy used in the clinical trial was exclusively composed of Alt a 1.

## 5. Conclusions

Immunotherapy with Alt a 1 desensitizes the treated patients, reducing their symptoms and medication consumption through the elimination of Alt a 1 sensitization, which is no longer present in the immunoblotting of some patients.

We conclude that almost all patients sensitized to *A. alternata* in Spain recognize the major allergen Alt a 1, specifically 98.4%. Of these, 70.3% only recognized the Alt a 1 allergen of all the allergens present in the allergenic extract of *A. alternata*.

In sensitization to *A. alternata,* there may be gender differences, with males being more likely to recognize more allergens than Alt a 1 in the *A. alternata* extract than females, and the latter being more responsive to immunotherapy treatment with Alt a 1.

## Figures and Tables

**Table 1:** Percentage of patients recognizing each of the allergens identified in the immunoblotting within each of the treatment groups of the clinical trial. P: placebo; Ld: low dose; Hd: high dose. Statistics are presented by analyzing the trial population by age group, gender, severity of rhinitis, and severity of asthma.

				Baseline Allergen Presence						Year 1 Allergen Presence						Year 2 Allergen Presence					
Item	Category (n)	Only Alt a 1	Group	Alt a 1	Alt a 3	Alt a 4/6	Alt a 7	Alt a 8	Alt a 10/15	Alt a 1	Alt a 3	Alt a 4/6	Alt a 7	Alt a 8	Alt a 10/15	Alt a 1	Alt a 3	Alt a 4/6	Alt a 7	Alt a 8	Alt a 10/15
	Total (64)	70.3% (45)	P	100	6.7	20	20	13.3	13.3	93.3	0	26.7	13.3	6.7	13.3	86.7	6.7	20	20	6.7	13.3
			Ld	100	0	25	15	15	10	90	0	35	25	25	15	80	0	35	35	35	20
			Hd	96.6	0	20.7	6.9	10.3	13.8	89.7	0	31.0	27.6	17.2	27.6	79.3	0	24.1	27.6	17.2	31
			Tot	98.4	1.6	21.9	12.5	12.5	12.5												
Age	Adolescents (26)	65.4% (17)	P	100	16.7	33.3	16.7	33.3	33.3	100	0	50 *	0 ^¥^	16.7	33.3 *	100	16.7	33.3	16.7	16.7	33.3 *
			Ld	100	0	25	12.5	12.5	25	100	0	37.5	25	37.5	25	75	0	37.5	50	50	37.5 ^¥^
			Hd	100	0	25	8.3	16.7	16.7	91.7	0	33.3	41.7 ^¥^	25	33.3	91.7	0	25	41.7 ^¥^	25	33.3
			Tot	100	3.8	26.9	11.5	19.2	23.1												
	Adults (38)	73.7% (28)	P	100	0	11.1	22.2	0	0	88.9	0	11.1	22.2 ^¥^	0	0	77.8	0	11.1	22.2	0	0
			Ld	100	0	25	16.7	16.7	0	83.3	0	33.3	25	16.7	8.3	83.3	0	33.3	25	25	8.3 ^¥^
			Hd	94.1	0	17.6	5.9	5.9	11.8	88.2	0	29.4	17.6 ^¥^	11.8	23.5	70.6	0	23.5	17.6 ^¥^	11.8	29.4
			Tot	97.4	0	18.4	13.2	7.9	5.3												
Gender	Male (37)	62.2% * (23)	P	100	10	30	10	20	20	90	0	40 ^¥^	0 ^¥^	10	20	90	10	30 ^¥^	10	10	20
			Ld	100	0	44.4	33.3	33.3	22.2	100	0	66.7 *	33.3	55.6 *	33.3 ^¥^	88.9	0	66.7 *	55.6	77.8 *	44.4 ^¥^
			Hd	100	0	27.8	11.1	16.7	16.7	94.4	0	44.4	33.3	27.8	38.9	88.9	0	38.9 ^¥^	33.3	27.8	44.4
			Tot	100	2.7	32.4 ^¥^	16.2	21.6	18.9												
	Female (27)	81.5% (22)	P	100	0	0	40	0	0	100	0	0 *	40 ^¥^	0	0	80	0	0 ^¥^	40 ^¥^	0	0
			Ld	100	0	9.1	0	0	0	81.8	0	9.1	18.2	0	0	72.7 *	0	9.1	18.2	0 *	0 ^¥^
			Hd	90.9	0	9.1	0	0	9.1	81.8	0	9.1 ^¥^	18.2	0	9.1	63.6 *	0	0 *	18.2	0	9.1
			Tot	96.3	0	7.4 *	7.4 ^¥^	0 *	3.7 *												
Rhinitis	Intermit. (6)	83.3% (5)	P	100	0	0	0	0	0	100	0	0	0	0	0	100	0	0	0	0	0
			Ld	100	0	0	0	0	0	100	0	0	0	0	0	100	0	0	100	0	0
			Hd	100	0	0	0	25	0	100	0	0	25	25	0	100	0	0	25	25	0
			Tot	100	0	0	0	16.7	0												
	Mild Persistent (13)	61.5% * (8)	P	100	0	50	0	50	50	100	0	50 ^¥^	0	50 *	50 *	100	50 *	50 *	50 *	50 *	50 *
			Ld	100	0	25	0	0	0	75.0	0	50 ^¥^	0	25	0	75.0	0	50	0 ^¥^	25	25
			Hd	85.7	0	28.6	0	0	14.3	85.7	0	42.9	28.6	0	57.1 ^¥^	71.4	0	42.9	28.6	0	57.1
			Tot	92.3	0	30.8	0	7.7	15.4												
	Mod/SevPersistent (45)	71.1% (32)	P	100	8.3	16.7	25	8.3	8.3	91.7	0	25	16.7	0	8.3	83.3	0	16.7	16.7	0	8.3
			Ld	100	0	26.7	20	20	13.3	93.3	0	33.3	33.3	26.7	20	80	0	33.3	40	40	20
			Hd	100	0	22.2	11.1	11.1	16.7	88.9	0	33.3	27.8	22.2	22.2	77.8	0	22.2	27.8	22.2	27.8
			Tot	100	2.2	22.2	17.8	13.3	13.3												
Asthma	Without asthma (22)	81.8% (18)	P	100	14.3	14.3	14.3	14.3	14.3	100	0	28.6	0	0	14.3	100	0	14.3	0	0	14.3
			Ld	100	0	16.7	0	0	0	100	0	50 ^¥^	16.7	16.7	16.7	100	0	50 ^¥^	16.7	33.3	33.3
			Hd	100	0	11.1	11.1	22.2	0	100	0	22.2	22.2	22.2	11.1	100	0	11.1	22.2	22.2	11.1
			Tot	100	4.5	13.6	9.1	13.6	4.5												
	Intermit. (16)	50%* (8)	P	100	0	100	0	100	100	100	0	100 *	0	100 *	100 *	100	100 *	100 *	100 *	100 *	100 *
			Ld	100	0	42.9	42.9	28.6	14.3	100	0	42.9	42.9	42.9	14.3	85.7	0	42.9	71.4 ^¥^	57.1	14.3
			Hd	87.5	0	25	0	0	25	87.5	0	37.5	37.5	12.5	25	87.5	0	25	37.5	12.5	37.5
			Tot	93.8	0	37.5	18.8	18.8	25												
	Mod/Sev Persistent (26)	73.1% (19)	P	100	0	14.3	28.6	0	0	85.7	0	14.3	28.6	0	0	71.4	0	14.3	28.6	0	0
			Ld	100	0	14.3	0	14.3	14.3	71.4	0	14.3	14.3	14.3	14.3	57.1 *	0	14.3 ^¥^	14.3	14.3	14.3
			Hd	100	0	25	8.3	8.3	16.7	83.3	0	33.3	25	16.7	41.7	58.3 *	0	33.3	25	16.7	41.7
			Tot	100	0	19.2	11.5	7.7	11.5												

* Statistically significant (*p* < 0.05) compared to the total population or ^¥^ compared to the opposite subgroup.

**Table 2:** Percentage of patients who improve in the efficacy parameters of the clinical trial, including band intensity for Alt a 1 on the immunoblotting performed, according to the assigned treatment group. Statistics are presented, analyzing the trial population by age group, gender, rhinitis severity, and asthma severity of the patients analyzed. S&M: symptoms and medication consumption score. IMB: immunoblotting. P: placebo; Ld: low dose; Hd: high dose.

			Patient Percentage Improvement Year 1						Patient Percentage Improvement Year 2				
Item	Category	Group	S&M	IMB	Wheal *Alternaria*	Wheal Alt a 1	IgE	IgG4	IMB	Wheal *Alternaria*	Wheal Alt a 1	IgE	IgG4
	Total	P	26.7 ^¥^	33.3 ^¥^	46.7	26.7 ^¥^	33.3	46.7	60	64.3	78.6	21.4	100
		Ld	45	60 ^¥^	70	90 ^¥^	26.3	89.5	70	80	90	31.6	89.5
		Hd	58.6 ^¥^	62.1 ^¥^	75.9	79.3 ^¥^	28.6	75.0	82.8	70.4	85.2	53.8	73.1
Allergen presence	Only Alt a 1	P	40 ^¥^	20 ^¥^	50	10 ^¥^	30	60 ^¥^	60	50	60	20	90
		Ld	46.7	60	66.7	100	20	93.3	66.7	80	93.3	33.3	93.3
		Hd	65	65	80	90	35.0	95	80	70	75.0	45	85
	More Allergens	P	0 ^¥^	60 ^¥^	40	60 ^¥^	40	20 ^¥^	60	46.8	100	20	100
		Ld	40	60	80	60	40	100	80	36.8 *	80	20	100
		Hd	62.5	50	62.5	50	12.5	87.5	100	44.9	87.5	62.5	100
Age	Adolescents	P	33.3	16.7 ^¥^	33.3	33.3	50	33.3	33.3	50	83.3	16.7	100
		Ld	50	62.5	62.5	87.5	25	100	62.5	75	75	25	100
		Hd	58.3	50	66.7	75	41.7 ^¥^	91.7	75	58.3	83.3	66.7	91.7
	Adults	P	22.2	44.4 ^¥^	55.6	22.2	22.2	55.6	77.8	66.7	66.7	22.2	88.9
		Ld	41.7	58.3	75	91.7	25	91.7	75	83.3	100	33.3	91.7
		Hd	64.7	64.7	82.4	82.4	17.6 ^¥^	94.1	88.2	70.6	76.5	35.3	88.2
Gender	Male	P	30	40	40	30	40	40	70	40 ^¥^	60 ^¥^	20	100
		Ld	55.6	33.3 ^¥^	66.7	77.8	11.1	100	44.4 ^¥^	77.8	77.8	11.1	100
		Hd	66.7	55.6	72.2	72.2	22.2	88.9	88.9	50	72.2	38.9	83.3
	Female	P	20	20	60	20	20	60	40	100 ^¥^	100 ^¥^	20	80
		Ld	36.4	81.8 ^¥^	72.7	100	36.4	90.9	90.9 ^¥^	81.8	100	45.5	90.9
		Hd	54.5	63.6	81.8	90.9	36.4	100	72.7	90.9 ^¥^	90.9	63.6 ^¥^	100
Rhinitis	Intermit.	P	100	0	0	0	100	0	0	0	0	0	100
		Ld	100	0	0	100	0	100	0	100	100	0	100
		Hd	75	75	75	75	25	100	75	50	75	50	75
	Mild Persistent	P	0	0	100	50	0	50	0	50	100	0	50
		Ld	50	75	50	100	25	75	75	75	75	50	75
		Hd	42.9	28.6	57.1	85.7	0 *	85.7	71.4	42.9	85.7	14.3 *	85.7
	Mod/Sev Persistent	P	25	41.7 ^¥^	41.7	25	33.3	50	75.0	66.7	75	25	100
		Ld	40	60	80	86.7	26.7	100	73.3	80	93.3	26.7	100
		Hd	66.7	66.7	83.3	77.8	38.9	94.4	88.9	77.8	77.8	61.1	94.4
Asthma	Without Asthma	P	42.9 ^¥^	28.6	28.6	14.3	57.1 ^¥^	57.1	57.1	42.9	57.1	14.3	85.7
		Ld	66.7 ^¥^	50	83.3	100	16.7	100	66.7	83.3	83.3	16.7	100
		Hd	44.4	66.7	77.8	77.8	22.2	100	88.9	33.3	66.7	33.3	100
	Intermit.	P	0 ^¥^	0 ^¥^	100	100 *	0 *	0 *	0 *	0 *	100	0	100
		Ld	42.9	42.9	57.1	71.4	14.3	100	57.1	71.4	85.7	14.3	100
		Hd	62.5	62.5	62.5	62.5	37.5	100	87.5	75.0	62.5	62.5	87.5
	Mod/Sev Persistent	P	14.3	42.9	57.1	28.6	14.3	42.9	71.4	85.7	85.7	28.6	100
		Ld	28.6	85.7	71.4	100	42.9	85.7	85.7	85.7	100	57.1 ^¥^	100
		Hd	75.0	50	83.3	91.7	25.0	91.7	75.0	75.0	91.7	41.7	91.7

* Statistically significant (*p* < 0.05) compared to the total population or ^¥^ compared to the opposite subgroup.

**Table 3:** Description of patients who developed Grade 1 and 2 systemic ADRs with their demographic and clinical characteristics, ADR term, and allergens present in the immunoblotting performed at baseline. S&M: symptoms and medication consumption score. M: male; F: female; P: placebo; Ld: low dose; Hd: high dose; MI: mild intermittent; SI: severe intermittent; MP: mild persistent; SP: moderate/severe persistent; WA: without asthma; I: intermittent; 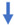: decrease at least 20%; 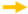: decrease less than 20%; 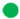: presence of allergen; 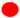: absence of allergen.

									IMBAlt a 1 Band		Baseline Allergen Presence					
#	Group	Age	Gender	Rhinitis	Asthma	Systemic ADR Grade 1	Systemic ADR Grade 2	S&M	Year 1	Year 2	Alt a 1	Alt a 3	Alt a 4/6	Alt a 7	Alt a 8	Alt a 10/15
2	Ld	14	M	SP	I	Dermatitis	No ADR	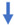	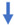	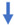	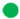	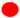	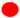	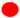	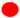	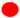
4	Ld	38	F	SP	SP	Rhinoconjunctivitis	No ADR	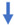	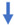	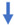	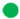	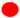	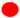	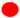	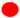	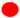
8	Hd	27	F	SP	SP	Headache Cough Rhinorrhea	Breathlessness	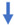	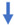	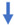	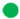	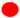	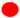	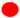	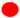	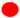
12	Hd	13	M	MP	SP	Pruritus	No ADR	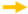	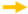	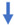	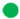	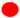	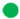	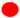	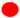	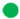
14	Hd	16	F	SP	SP	Presyncope	No ADR	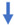	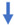	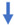	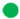	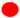	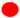	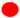	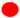	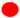
27	Ld	13	M	I	I	Presyncope	No ADR	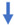	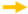	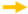	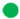	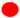	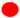	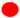	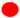	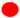
34	P	12	M	I	WA	Torticulis	No ADR	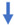	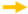	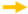	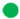	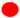	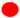	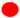	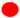	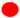
39	Ld	12	M	SP	I	Contusion Respiratory infection	No ADR	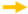	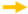	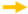	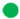	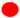	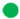	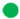	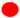	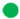
49	Ld	28	M	SP	I	Sinusitis	No ADR	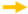	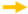	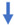	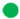	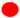	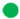	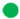	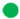	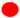
50	Hd	33	M	SP	WA	Rhinitis	No ADR	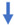	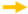	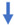	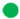	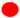	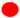	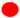	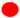	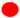
56	P	35	F	SP	SP	Itching	No ADR	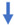	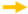	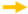	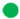	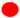	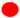	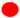	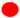	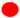
64	Ld	42	F	MP	MP	Rhinitis	Asthma	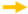	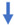	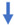	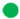	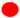	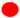	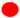	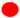	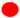

## Data Availability

The authors have ensured that the data shared are in accordance with the consent provided by participants on the use of confidential data, following Reglamento (UE) 2016/679 del Parlamento europeo y del Consejo de 27 de abril de 2016 de Protección de Datos (RGPD) and good clinical practice and the Declaration of Helsinki. The data presented in this study are available on request from the corresponding author. The data are not publicly available due to GDPR.

## Supplementary Materials

The following are available online at https://www.mdpi.com/article/10.3390/jof7110974/s1, Table S1: Demographic and clinical data of the population participating in the clinical trial of immunotherapy with Alt a 1, Table S2: Description of allergens present in immunoblotting per patient.
