# Supplementary material for: Changes in the Sensitization Pattern to Alternaria alternata Allergens in Patients Treated with Alt a 1 Immunotherapy
Source: J Fungi (Basel). 2021 Nov 16;7(11):974. doi: 10.3390/jof7110974 (PMC8618185; doi:10.3390/jof7110974)
Supplement: Supplementary file 1 [file jof-07-00974-s001.zip › Table S1.zip!/Table S1.pdf]

**Table S1.** Summary of the demographic data of the population analysed in this study, already analysed in the previous cliical trial (J Allergy Clin Immunol 2019;144:216-23). P: Placebo; Ld: Low dose; Hd: High dose; MI: Mild Intermittent; SI: Severe Intermittent; MP: Mild Persistent; SP: Moderate/Severe Persistent; WA: Without asthma; I: Intermittent.

| | | | | **Rhinitis (%)** | | | | **Asthma (%)** | | | | **Immunoglobulins** | | **Wheal area** | |
|---|---|---|---|---|---|---|---|---|---|---|---|---|---|---|---|
| **Group** | **Age (Mean/Median)** | **<18 (%)** | **Males (%)** | **MI** | **SI** | **MP** | **SP** | **WA** | **I** | **MP** | **SP** | **IgE** | **IgG4** | ***A. alternata*** | **Alt a 1** |
| **P** | 23.4/21 | 66.7 | 40 | 0 | 6.7 | 13.3 | 80 | 46.7 | 6.7 | 20 | 26.7 | 10.5 | 36.5 | 46.1 | 51.5 |
| **Ld** | 22.7/22 | 45 | 40 | 0 | 5 | 20 | 75 | 30 | 35 | 20 | 15 | 14.2 | 30.5 | 43.5 | 50.1 |
| **Hd** | 20.4/18 | 62.1 | 41.4 | 3.4 | 10.3 | 24.1 | 62.1 | 31 | 27.6 | 24.1 | 17.2 | 15.9 | 63.9 | 43.3 | 41.6 |
| **Total** | 21.7/19.5 | 57.8 | 40.6 | 1.6 | 7.8 | 20.3 | 57.8 | 34.4 | 25 | 21.9 | 18.8 | 15 | 50.5 | 43.8 | 46.4 |
