# Supplementary material for: Changes in the Sensitization Pattern to Alternaria alternata Allergens in Patients Treated with Alt a 1 Immunotherapy
Source: J Fungi (Basel). 2021 Nov 16;7(11):974. doi: 10.3390/jof7110974 (PMC8618185; doi:10.3390/jof7110974)
Supplement: Supplementary file 1 [file jof-07-00974-s001.zip › Table S2.zip!/Table S2.pdf]

Table S2. Description of allergens present in immunoblotting per patient. Mon: monosensitized; Poly: Polysensitized; P: Placebo; Ld: Low dose; Hd: High dose; 🟢:presence of allergen; 🔴:absence of allergen.

			Allergen Presence Baseline						Allergen Presence Year 1						Allergen Presence Year 2					
#	Group	Allergens	Alt a 1	Alt a 3	Alt a 4/6	Alt a 7	Alt a 8	Alt a 10/15	Alt a 1	Alt a 3	Alt a 4/6	Alt a 7	Alt a 8	Alt a 10/15	Alt a 1	Alt a 3	Alt a 4/6	Alt a 7	Alt a 8	Alt a 10/15
1	Hd	Mon	🟢	🔴	🔴	🔴	🔴	🔴	🔴	🔴	🟢	🔴	🟢	🟢	🔴	🔴	🟢	🔴	🟢	🟢
2	Ld	Mon	🟢	🔴	🔴	🔴	🔴	🔴	🟢	🔴	🔴	🔴	🔴	🔴	🔴	🔴	🔴	🟢	🟢	🔴
3	Hd	Poly	🟢	🔴	🟢	🟢	🟢	🟢	🟢	🔴	🟢	🟢	🟢	🟢	🟢	🔴	🟢	🟢	🟢	🟢
4	Ld	Mon	🟢	🔴	🔴	🔴	🔴	🔴	🟢	🔴	🔴	🔴	🔴	🔴	🟢	🔴	🔴	🔴	🔴	🔴
5	Hd	Mon	🟢	🔴	🔴	🔴	🔴	🔴	🟢	🔴	🔴	🔴	🔴	🔴	🟢	🔴	🔴	🔴	🔴	🔴
6	Ld	Poly	🟢	🔴	🟢	🔴	🔴	🔴	🟢	🔴	🟢	🔴	🔴	🔴	🟢	🔴	🟢	🔴	🔴	🔴
7	Hd	Mon	🟢	🔴	🔴	🔴	🔴	🔴	🟢	🔴	🔴	🔴	🔴	🔴	🟢	🔴	🔴	🔴	🔴	🔴
8	Hd	Mon	🟢	🔴	🔴	🔴	🔴	🔴	🟢	🔴	🔴	🔴	🔴	🔴	🔴	🔴	🔴	🔴	🔴	🔴
9	Hd	Mon	🟢	🔴	🔴	🔴	🔴	🔴	🟢	🔴	🔴	🔴	🔴	🔴	🔴	🔴	🔴	🔴	🔴	🔴
10	P	Mon	🟢	🔴	🔴	🔴	🔴	🔴	🟢	🔴	🔴	🔴	🔴	🔴	🟢	🔴	🔴	🔴	🔴	🔴
11	P	Mon	🟢	🔴	🔴	🔴	🔴	🔴	🟢	🔴	🔴	🔴	🔴	🔴	🟢	🔴	🔴	🔴	🔴	🔴
12	Hd	Poly	🟢	🔴	🟢	🔴	🔴	🟢	🟢	🔴	🔴	🟢	🔴	🟢	🟢	🔴	🔴	🟢	🔴	🟢
13	Ld	Mon	🟢	🔴	🔴	🔴	🔴	🔴	🟢	🔴	🔴	🟢	🔴	🔴	🟢	🔴	🔴	🟢	🔴	🔴
14	Hd	Mon	🟢	🔴	🔴	🔴	🔴	🔴	🔴	🔴	🔴	🔴	🔴	🔴	🔴	🔴	🔴	🔴	🔴	🔴
15	Ld	Mon	🟢	🔴	🔴	🔴	🔴	🔴	🟢	🔴	🔴	🔴	🔴	🔴	🔴	🔴	🔴	🔴	🔴	🔴
16	P	Poly	🟢	🔴	🟢	🔴	🔴	🔴	🔴	🔴	🟢	🔴	🔴	🔴	🔴	🔴	🟢	🔴	🔴	🔴
17	P	Mon	🟢	🔴	🔴	🔴	🔴	🔴	🟢	🔴	🔴	🔴	🔴	🔴	🟢	🔴	🔴	🔴	🔴	🔴
18	Hd	Mon	🟢	🔴	🔴	🔴	🔴	🔴	🟢	🔴	🔴	🔴	🔴	🔴	🟢	🔴	🔴	🔴	🔴	🔴
19	Hd	Mon	🟢	🔴	🔴	🔴	🔴	🔴	🟢	🔴	🔴	🔴	🔴	🔴	🟢	🔴	🔴	🔴	🔴	🔴
20	P	Mon	🟢	🔴	🔴	🔴	🔴	🔴	🟢	🔴	🔴	🔴	🔴	🔴	🟢	🔴	🔴	🔴	🔴	🔴
21	Hd	Mon	🟢	🔴	🔴	🔴	🔴	🔴	🟢	🔴	🔴	🔴	🔴	🔴	🟢	🔴	🔴	🔴	🔴	🔴
22	Hd	Poly	🟢	🔴	🟢	🔴	🔴	🔴	🟢	🔴	🟢	🔴	🔴	🔴	🟢	🔴	🔴	🔴	🔴	🔴
23	Ld	Poly	🟢	🔴	🟢	🔴	🟢	🟢	🟢	🔴	🟢	🔴	🟢	🟢	🟢	🔴	🟢	🔴	🟢	🟢
24	Hd	Poly	🟢	🔴	🟢	🔴	🔴	🟢	🟢	🔴	🟢	🔴	🔴	🟢	🟢	🔴	🔴	🔴	🔴	🟢
25	Hd	Mon	🟢	🔴	🔴	🔴	🔴	🔴	🟢	🔴	🔴	🔴	🔴	🔴	🟢	🔴	🔴	🔴	🔴	🔴
26	Hd	Mon	🟢	🔴	🔴	🔴	🔴	🔴	🟢	🔴	🔴	🟢	🔴	🔴	🟢	🔴	🔴	🟢	🔴	🔴
27	Ld	Mon	🟢	🔴	🔴	🔴	🔴	🔴	🟢	🔴	🔴	🔴	🔴	🔴	🟢	🔴	🔴	🟢	🔴	🔴
28	P	Poly	🟢	🔴	🟢	🔴	🟢	🟢	🟢	🔴	🟢	🔴	🟢	🟢	🟢	🟢	🟢	🟢	🟢	🟢
29	Ld	Mon	🟢	🔴	🔴	🔴	🔴	🔴	🟢	🔴	🟢	🔴	🟢	🔴	🟢	🔴	🟢	🔴	🟢	🟢
30	Hd	Mon	🟢	🔴	🔴	🔴	🔴	🔴	🟢	🔴	🔴	🟢	🔴	🔴	🟢	🔴	🔴	🟢	🔴	🔴
31	Ld	Mon	🟢	🔴	🔴	🔴	🔴	🔴	🟢	🔴	🔴	🟢	🔴	🔴	🟢	🔴	🔴	🟢	🔴	🔴
32	Ld	Mon	🟢	🔴	🔴	🔴	🔴	🔴	🟢	🔴	🔴	🔴	🔴	🔴	🟢	🔴	🔴	🔴	🔴	🔴
33	P	Mon	🟢	🔴	🔴	🔴	🔴	🔴	🟢	🔴	🟢	🔴	🔴	🟢	🟢	🔴	🟢	🔴	🔴	🟢
34	P	Mon	🟢	🔴	🔴	🔴	🔴	🔴	🟢	🔴	🔴	🔴	🔴	🔴	🟢	🔴	🔴	🔴	🔴	🔴
35	Ld	Mon	🟢	🔴	🔴	🔴	🔴	🔴	🟢	🔴	🔴	🔴	🔴	🔴	🟢	🔴	🔴	🔴	🔴	🔴
36	Hd	Poly	🟢	🔴	🔴	🔴	🔴	🟢	🟢	🔴	🟢	🟢	🟢	🟢	🟢	🔴	🟢	🟢	🟢	🟢
37	P	Mon	🟢	🔴	🔴	🔴	🔴	🔴	🟢	🔴	🔴	🔴	🔴	🔴	🟢	🔴	🔴	🔴	🔴	🔴
38	Hd	NR	🔴	🔴	🔴	🔴	🔴	🔴	🔴	🔴	🔴	🔴	🔴	🔴	🔴	🔴	🔴	🔴	🔴	🔴
39	Ld	Poly	🟢	🔴	🟢	🟢	🔴	🟢	🟢	🔴	🟢	🟢	🟢	🟢	🟢	🔴	🟢	🟢	🟢	🟢
40	P	Poly	🟢	🔴	🔴	🟢	🔴	🔴	🟢	🔴	🔴	🟢	🔴	🔴	🔴	🔴	🔴	🟢	🔴	🔴
41	Hd	Mon	🟢	🔴	🔴	🔴	🔴	🔴	🟢	🔴	🔴	🟢	🔴	🔴	🟢	🔴	🟢	🟢	🔴	🟢
42	Ld	Mon	🟢	🔴	🔴	🔴	🔴	🔴	🟢	🔴	🟢	🔴	🔴	🟢	🟢	🔴	🟢	🔴	🟢	🟢
43	Ld	Mon	🟢	🔴	🔴	🔴	🔴	🔴	🔴	🔴	🔴	🔴	🔴	🔴	🔴	🔴	🔴	🔴	🔴	🔴
44	Hd	Mon	🟢	🔴	🔴	🔴	🔴	🔴	🟢	🔴	🔴	🔴	🔴	🔴	🟢	🔴	🔴	🔴	🔴	🔴
45	Ld	Mon	🟢	🔴	🔴	🔴	🔴	🔴	🔴	🔴	🔴	🔴	🔴	🔴	🔴	🔴	🔴	🔴	🔴	🔴
46	P	Mon	🟢	🔴	🔴	🔴	🔴	🔴	🟢	🔴	🔴	🔴	🔴	🔴	🟢	🔴	🔴	🔴	🔴	🔴
47	Hd	Poly	🟢	🔴	🔴	🔴	🟢	🔴	🟢	🔴	🔴	🔴	🟢	🔴	🟢	🔴	🔴	🔴	🟢	🔴
48	Hd	Poly	🟢	🔴	🟢	🟢	🟢	🔴	🟢	🔴	🟢	🟢	🟢	🔴	🟢	🔴	🔴	🟢	🟢	🔴
49	Ld	Poly	🟢	🔴	🟢	🟢	🟢	🔴	🟢	🔴	🟢	🟢	🟢	🔴	🟢	🔴	🟢	🟢	🟢	🔴
50	Hd	Mon	🟢	🔴	🔴	🔴	🔴	🔴	🟢	🔴	🔴	🔴	🔴	🔴	🟢	🔴	🔴	🔴	🔴	🔴
51	Hd	Mon	🟢	🔴	🔴	🔴	🔴	🔴	🟢	🔴	🔴	🔴	🔴	🔴	🟢	🔴	🔴	🔴	🔴	🔴
52	Hd	Mon	🟢	🔴	🔴	🔴	🔴	🔴	🟢	🔴	🟢	🔴	🔴	🟢	🔴	🔴	🟢	🔴	🔴	🟢
53	Ld	Mon	🟢	🔴	🔴	🔴	🔴	🔴	🟢	🔴	🔴	🔴	🔴	🔴	🟢	🔴	🔴	🔴	🔴	🔴
54	Hd	Mon	🟢	🔴	🔴	🔴	🔴	🔴	🟢	🔴	🟢	🔴	🔴	🟢	🟢	🔴	🟢	🔴	🔴	🟢
55	Ld	Poly	🟢	🔴	🟢	🟢	🟢	🔴	🟢	🔴	🟢	🟢	🟢	🔴	🟢	🔴	🟢	🟢	🟢	🔴
56	P	Mon	🟢	🔴	🔴	🔴	🔴	🔴	🟢	🔴	🔴	🔴	🔴	🔴	🟢	🔴	🔴	🔴	🔴	🔴
57	P	Mon	🟢	🔴	🔴	🔴	🔴	🔴	🟢	🔴	🔴	🔴	🔴	🔴	🟢	🔴	🔴	🔴	🔴	🔴
58	Ld	Mon	🟢	🔴	🔴	🔴	🔴	🔴	🟢	🔴	🔴	🔴	🔴	🔴	🟢	🔴	🔴	🔴	🔴	🔴
59	P	Poly	🟢	🔴	🔴	🟢	🔴	🔴	🟢	🔴	🔴	🟢	🔴	🔴	🟢	🔴	🔴	🟢	🔴	🔴
60	P	Poly	🟢	🟢	🟢	🟢	🟢	🟢	🟢	🔴	🟢	🔴	🔴	🔴	🟢	🔴	🔴	🔴	🔴	🔴
61	Hd	Mon	🟢	🔴	🔴	🔴	🔴	🔴	🟢	🔴	🔴	🔴	🔴	🔴	🟢	🔴	🔴	🔴	🔴	🔴
62	Hd	Poly	🟢	🔴	🟢	🔴	🔴	🔴	🟢	🔴	🟢	🟢	🔴	🟢	🟢	🔴	🟢	🟢	🔴	🟢
63	Hd	Mon	🟢	🔴	🔴	🔴	🔴	🔴	🟢	🔴	🔴	🔴	🔴	🔴	🟢	🔴	🔴	🔴	🔴	🔴
64	Ld	Mon	🟢	🔴	🔴	🔴	🔴	🔴	🟢	🔴	🔴	🔴	🔴	🔴	🟢	🔴	🔴	🔴	🔴	🔴
